# Genetic Parameter Estimation and Selection for Resistance to Gastrointestinal Nematode Parasites in Sheep—A Review

**DOI:** 10.3390/ani14040613

**Published:** 2024-02-14

**Authors:** Samla Marques Freire Cunha, Olivia Willoughby, Flavio Schenkel, Ángela Cánovas

**Affiliations:** Centre for Genetic Improvement of Livestock, Department of Animal Biosciences, University of Guelph, 50 Stone Rd E, Guelph, ON N1G 2W1, Canada; scunha@uoguelph.ca (S.M.F.C.); owilloug@uoguelph.ca (O.W.); schenkel@uoguelph.ca (F.S.)

**Keywords:** breeding programs, genetics, genomics, GIN, parasite, resistance, sheep

## Abstract

**Simple Summary:**

Gastrointestinal nematode (GIN) infection in sheep is a common problem affecting small and large farms worldwide. These GIN parasites will normally infect the intestine of a sheep and may lead to diarrhea, weight loss, anemia and, in some cases, the animal’s death. The animals can also present a decrease in meat, wool, and milk production, which will lead to substantial economic losses. The use of anthelmintics to treat infected sheep as a single approach has resulted in worms developing resistance against the available commercial drugs. Other studies have shown that the animals resistant to GIN infection are capable of coping with the infection and have reduced health impairment caused by the GIN parasites. In this review, we summarize the results from the literature on the estimated genetic parameters for traits assessing GIN resistance in sheep. Additionally, we summarize the possible genetic gains when sheep are selected to be resistant to GIN and how genetics and genomics have impacted breeding programs in different countries.

**Abstract:**

Gastrointestinal nematodes (GINs) are a major problem affecting sheep production systems worldwide. The flocks infected with GINs can undergo significant economic losses due to a decrease in productivity, the animals’ deaths, and the costs associated with treatments. The over-reliance on anthelmintics in the past years to eliminate GINs has resulted in the development of resistance against the available commercial anthelmintics. Genetically resistant animals can be used in mating systems to improve the overall flock resistance. This review aimed to summarize the estimated genetic parameters for resistance traits and genetic gains through the use of genetic/genomic selection for resistance to GINs in sheep. Heritability estimates from the literature ranged from 0.00 to 0.46 for fecal egg counts, 0.12 to 0.37 for packed cell volume/hematocrit, 0.07 to 0.26 for FAffa MAlan CHArt (FAMACHA©), from 0.10 to 0.37 for blood parameters, and 0.19 for Immunoglobulin A. Genetic correlations between traits measuring resistance to GINs and production traits ranged from negative to positive values in the literature. Genetic gains are possible when genetic/genomic selection for GIN resistance is applied. Therefore, genetic/genomic selection can be used to improve flocks’ resistance to GINs as a sustainable approach in sheep production systems.

## 1. Introduction

Sheep production systems worldwide are at risk of gastrointestinal nematode (GIN) infection, which is a common and detrimental parasitic infection affecting sheep [1,2]. In sheep, the principal GIN species that infect the animals include *Haemonchus contortus*, *Teladorsagia circumcincta*, *Trichostrongylus* spp. (predominantly *Trichostrongylus colubriformis*), *Cooperia curticei*, and *Oesophagostomum* spp. [3,4]. Other species of GIN that can infect sheep include *Nematodirus* spp., *Trichuris ovis*, *Bunostomum trigonocephalum*, and *Strongyloides papillosus*, but these species have less economic and clinical importance [3]. The level of importance of a GIN and its ability to produce clinical signs when infecting an animal will vary based on the geographical location, mainly due to weather differences among locations [3].

The distribution of GIN species varies across different regions around the world due to mainly the weather conditions [3]. For instance, Gutiérrez-Gil et al. [5] identified *Teladorsagia* sp. (65.5%) and *Trichostrongylus* spp. (30.5%) as the most prevalent genera in the Spanish Churra sheep breed in Spain. In farms in the United States, Kaplan et al. [6] identified *H. contortus* (91.0%) as the prevalent species, followed by *Trichostrongylus* spp. (9.0%). In a study performed in Canada by Mederos et al. [7], *Teladorsagia* sp. was the most common species in the first year of the study, followed by *H. contortus*, and in the second year of the study, *H. contortus* was the most common species, followed by *Teladorsagia* sp. Mederos et al. [7] also pointed out that differences in the prevalence of *H. contortus* between two provinces included in the study (Ontario and Quebec) could be due to the different levels of rainfall. In Limpopo Province, South Africa, *H. contortus* was the most prevalent GIN identified, followed by *Teladorsagia*/*Trichostrongylus* and *O. columbianum* [8]. Santos et al. [9] identified *Haemonchus*, *Trichostrongylus*, *Cooperia*, and *Oesophagostomum* as the most common genera in both the dry and rainy seasons in Brazil. In Malaysia, Mursyidah et al. [10] identified *H. contortus* (75%), *Trichostrongylus* sp. (24%), and *Oesophagostomum* sp. (1%) as the most common GINs infecting small ruminants.

Gastrointestinal nematodes can negatively affect the health and welfare of the infected animals. Worms will infect the abomasum and small intestine of small ruminants, which can lead to the development of clinical signs of infection [3]. Animals can present signs of anemia, hypoproteinemia, depression, loss of body condition, reduced production, weight loss, a decreased appetite, and sometimes, death [6,11]. Additionally, animals with GIN infection are associated with high levels of cortisol and may experience decreased animal welfare [12]. Gastrointestinal nematodes are also responsible for massive economic losses in the sheep industry [5,11]. It has been estimated that these parasites cause losses of tens of billions of dollars globally based on the sales of anti-parasitic drugs by pharmaceutical companies alone, which does not take into consideration the significant loss in productivity that can result from these infections [13]. Two main expenses can be expected due to GIN infection. First, there are expenses related to infection control, such as the purchase of anthelmintics, veterinary care, and management. Secondly, there are expenses related to the decrease in growth rate, increase in mortality rate and, ultimately, reduction in production (e.g., meat, milk, and wool production) [5]. McLeod [14] and Sackett [15] investigated the economic losses associated with GIN in sheep production systems and stated that the majority of the economic losses are associated with a decrease in production instead of cost of treatments. Although it is possible to treat infected animals with anthelmintics, the over-reliance on them to eliminate GINs and their repeated misuse over time culminated in the development of anthelmintic resistance against the few available commercial drugs in most GIN populations worldwide [16,17,18]. In addition, increased concerns about food safety and environmental contamination have magnified the need to search for alternatives to reduce anthelmintic use [16,17,19].

The identification of animals resistant to GIN infection for use in breeding strategies is not an original idea. Natural selection happens in wild populations, where the resistant animals survive and mate, generating offspring capable of dealing with infections [20]. There are known sheep breeds that are naturally resistant to GINs all around the world, including the Santa Inês [21], Morada Nova [22], Katahdin [23,24], St. Croix [23,24], Gulf Coast Native [24], Florida Native [24], Red Massai [25], Barbados Black Belly [24,25], and others. Among the susceptible sheep breeds, several studies demonstrated genetic variation within them, making it possible to select for more resistant animals [2,26]. Additionally, studies have also estimated moderate heritabilities for traits assessing resistance, showing that it is possible to select these traits to improve the resistance to GINs in sheep [12,27,28].

This review aims to summarize and discuss the genetic parameters associated with resistance against GIN, the genetic and genomic selection results over the years, and the possible genetic gains achieved from the selection for GIN resistance.

## 2. Resistance and Resilience

To further discuss the genetic parameters and genetic gains associated with selection for resistance to GINs in sheep, it is important to understand the difference between resistance and resilience and how these phenotypes can be measured. Bishop and Stear [20] defined resistance as the individual ability of an animal to “resist infection or control the parasite life cycle” [29]. In other words, it is the animal’s capacity “to prevent or limit the proliferation of the pathogen once it is infected, the ability of the animal to expel the pathogen, or the ability of the animal to limit the shedding of infective material”. Regarding the ambit of nematode infection, this could include the probability that the ingested larvae will be established, the probability that the parasite will develop itself inside the host, and nematode mortality and fecundity [30]. On the other hand, resilience is the animal’s ability to keep its productive levels regardless of parasitic infection [20,31].

Different phenotypes can be used to measure resistance and resilience to GINs, and it is recommended to evaluate the animals in an environment of a nematode challenge, which can happen under natural or artificial conditions [30]. The phenotypes that measure resistance and resilience are normally related to either the animal’s immune response or productivity levels in the face of an infection. The number, size, and fecundity of the parasites found in the abomasum or small intestine, faecal egg count (FEC), hematocrit (HCT), packed cell volume (PCV), eosinophilia, immunoglobulin (Ig) level (e.g., IgA, IgM, and IgG), dag score, and FAffa MAlan CHArt (FAMACHA©) score are examples of traits that measure resistance [18,30,32]. On the other hand, resilience can be measured as growth rate, body condition score (BCS), and need for treatment [30].

The trait most commonly used as a measurement of GIN resistance in sheep is FEC [18,30,31]. This trait is heritable, repeatable, and a reliable parameter to measure the animals’ resistance [17,33]. The number of eggs counted in the faeces is used as an estimate for the number of female worms within the host and the worm’s fecundity capacity [17,31]. Animals with low FEC values are desired. Selecting for resistance using FEC is favorable since resistant animals shed fewer eggs in the faeces, demonstrating a diminishing pasture infestation rate [20,30,34]. Fewer larvae available on pasture will decrease the number of individual challenges and reduce the overall FEC, with a positive impact on production and the number of anthelmintic treatments required [20,30,34]. Even when the FEC only considers a specific species, selecting for one species could provide resistance to different species, though at a lower level than the primary species being selected against [19].

Despite FEC being a very commonly used trait to measure resistance, some limitations can be pointed out. Differences in the final egg count can happen due to the use of different methodologies, changes in the faeces composition since FEC is a ratio (eggs per gram of faeces) [27], infections with multiple species due to different fertility levels [18,27], different proportions of male and female worms infecting the animals, and the impossibility of differentiating the eggs for each species [17]. The FEC also can vary over time, which may require repeated measurements, becoming impractical and time consuming for producers [17,18,35]. Another drawback of FEC is that the animals need to be infected with GINs to be measured, and treatments with anthelmintics will inhibit the true egg counts, misleading the animal’s true resistance [17,18].

Another phenotype commonly associated with GIN parasitemia is FAMACHA©. This measurement relies on a subjective scoring system, from one (red) to five (pale/white), of the ocular conjunctival mucous membrane colour, which can be indicative of anemia [6,11]. The advantages of using FAMACHA© scores include the ease of measurement on a farm without the need for specific equipment, and the fact that it can be performed during usual farm management. Using FAMACHA© can also allow the producers to practice targeted selective deworming, in which they only treat animals that they suspect are heavily infested with parasites, reducing the number of animals that need to be dewormed and avoiding the development anthelmintic resistance [17,27]. However, anemia is not a sign exclusive to GIN infection, as other diseases can cause this clinical sign to be present [17,35,36]. Furthermore, the FAMACHA© scores are highly related to *H. contortus* infection due to its hematophagous characteristic, which makes the detection of infections by other GIN species difficult [17,35]. However, in flocks with predominantly *H. contortus* infection, FAMACHA© could be used as a trait to select for resistant animals [37].

Another trait used to measure the level of anemia is PCV, also known as HCT [32]. This trait is defined as the ratio of red blood cell volume to the total volume of blood [32] and has been used largely in studies about resistance/resilience to GINs in sheep. This can be a very helpful parameter in infections, where all or most of the parasites are blood-sucking worms [17]. However, this trait can be invasive to measure in an animal due to blood collection and may be cost- and time-prohibitive due to the need for trained personnel to perform the analysis.

Host immune response can also be used to measure an animal’s resistance/resilience to GINs. However, the components of this response may change depending on the infective species and the stage of infection within the same species [17]. Immunoglobulin levels (e.g., IgA, IgE, and IgG) can be useful to understand the immunological mechanisms that differentiate resistant and susceptible animals. Although the animals need to be exposed to GINs in order to produce immunoglobins against them, this is a direct and specific measurement of the immune response to GIN infection [17,18]. Additionally, immunoglobulin production is not affected by anthelmintic usage, and it is possible to identify a specific time to measure Ig levels depending on the region or climate the animals are living in [18,35]. Measuring Ig levels can be expensive and laborious, as it is necessary a laboratory structure to analyze the collected material [17]. Other immune markers can also be used to measure an animal’s response to GINs, such as mast cells, globule leucocytes, and tissue eosinophils [17].

There are a variety of other traits that can measure resistance/resilience to GINs. These traits also present positive and negative aspects. Traits such as worm count, length, and fecundity are very precise for assessing host resistance, can identify different species, can confirm the FEC results, and are important for understanding the host immune responses for individual GIN species. However, these measurements can only be performed post mortem, are costly and laborious, and are not routinely used [17]. Plasma protein levels and albumin levels can also be used to measure host resistance due to the parasites’ ability to provoke hypoproteinemia [17]. These measurements can be important to determine protein levels and to confirm suspicions of worm infection. However, it is economically costly and laborious to collect and analyze them [17]. The accumulation of faeces in the wool in the breech area, known as “dag score,” can be measured and can be indicative of diarrhea. However, diarrhea is not a sign exclusive to GIN infection and can present itself in other diseases [35,36]. Body weight (BW) and BCS can be used to measure resilience to GIN infection. The advantages of using BCS and BW are that these traits can be easily measured on a farm, with no need for special equipment in addition to a body weight scale, and they are often routinely measured as a normal part of flock management [27]. However, both traits are affected by factors that are not related to GIN infection, including nutrition. Furthermore, body weight differences between resistant/resilient and susceptible animals can be small or insignificant, making it hard to differentiate between them [17].

## 3. Genetic Parameters for Traits Measuring Resistance/Resilience

To be able to improve a trait through genetic selection, it needs to be heritable and show genetic variation in the population. Studies have shown that FEC [5,22,27,28,32,38], FAMACHA© score [27], PCV [28,32], Ig levels [5], and other traits [5,22,32] used to measure resistance in sheep are heritable, with genetic and phenotypic variations among breeds and individuals, enabling the use of these traits in genetic selection programs [1]. In addition, it is also important to understand how resistance/resilience correlates to the production traits, making possible to optimally select for different traits in breeding programs [39].

There are several studies estimating the genetic parameters for several traits measuring resistance/resilience in sheep. These studies differ in terms of several aspects, such as their objectives, breeds, methodology used, location, type of challenge, phenotypes, age, and the sex of the animal, among others. The estimation of reliable genetic parameters relies on the sample size, which should be large enough [40,41], but this can be difficult to find for resistance to GIN traits due to the cost and labour needed to collect the phenotypes over many successive years. This is of particular importance for breeding programs in the initial phases of applying selection to resistance to GIN. It is important to exercise caution when using literature estimates of genetic parameters in the genetic evaluation for GIN resistance traits, since these estimates can vary significantly between studies [1].

### 3.1. Heritabilities Estimates for Traits Measuring Resistance/Resilience

Recently, three meta-analysis studies on genetic parameters for several traits, including resistance and production traits, were published [1,39,42]. Hayward [1] estimated a global heritability using multi-level analysis of 0.25 for resistance to GIN from 121 studies. According to the author, the heritability ($h^{2}$) estimates were not significantly influenced by latitude, age, infection type, statistical method, and maternal effects. Nevertheless, the estimates were influenced by sex, the trait evaluated, and parasite species. Studies with only males had smaller $h^{2}$ estimates than the studies with only females or mixed sexes. Worm length, number, and fecundity presented smaller $h^{2}$ estimates when compared with the FEC, FAMACHA© scores, and parasite-specific antibody response. Infections where *Nematodirus* spp. and *Trichostrongylus* spp. were the prevalent species showed smaller $h^{2}$ estimates when compared with the infections mainly caused by *H. contortus* and *T. circumcincta*. The heritability estimates for different studies and traits are summarized in Table 1.

Investigating the heritability estimates for FEC in their meta-analysis, Medrado et al. [42] estimated a combined $h^{2}$ using the DerSimonian and Laird Method of 0.17 for FEC using 37 studies. Meanwhile, Mucha et al. [39] reported pooled $h^{2}$ estimates of 0.14 (±0.04) and 0.29 (±0.03) for FEC in dairy sheep and meat sheep, respectively. Mucha et al. [39] clustered the estimations obtained in a similar condition by using the Hierarchical Ward clustering method and performed meta-analysis using a three-level model using the restricted maximum likelihood. An earlier literature review was performed by Safari et al. [40], and a weighted mean $h^{2}$ of 0.27 (±0.02) for FEC was estimated from 16 studies.

Taking a closer look at the studies that estimated $h^{2}$ for FEC under natural grazing conditions, Oliveira et al. [43] estimated an $h^{2}$ value of 0.19 (±0.03) for FEC in the Santa Inês sheep breed in Brazil. Boareki et al. [27] estimated $h^{2}$ values of 0.10 (±0.04) and 0.07 (±0.05) for the transformed FEC, measured with the Modified McMaster and Triple Chamber McMaster methods, respectively. When both measurements were integrated, the estimate of $h^{2}$ was 0.12 (±0.04). Gutiérrez-Gil et al. [5] estimated $h^{2}$ values for FEC of 0.12 (±0.04) on the day of deworming and 0.09 (±0.03) 60 days after deworming, with natural exposure during grazing. Gutiérrez-Gil et al. [5] estimated a significant genetic correlation between both the estimates of 0.82 (±0.11) and Boareki et al. [27] estimated a genetic correlation of 0.94 (±0.17) between both FEC $h^{2}$ estimates using the Modified McMaster and Triple Chamber McMaster methods. Khusro et al. [16] estimated $h^{2}$ values for FEC measured at 12 and 16 months of 0.21 (±0.02) and 0.38 (±0.03), respectively, under a natural challenge condition.

Hollema et al. [12] considered different environments of worm challenges using natural exposure and a bivariate model to estimate $h^{2}$ values for FEC of 0.19 (±0.03) and 0.37 (±0.03) for the low- and high-burdenenvironments, respectively, with an overall $h^{2}$ of 0.20 (±0.03). According to the authors, the genetic correlation for FEC in the low- and high-burdenenvironment was 0.30 (±0.16), indicating the re-ranking of sires. Hollema et al. [12] also applied a random regression model to the data and estimated $h^{2}$ values of 0.25, 0.25, and 0.36 for the low-, medium-, and high-burden environments, respectively. The authors estimated a genetic correlation of 0.24 between the high- and low-burden environments, indicating large-scale animal re-ranking between both the environments. According to the authors, the estimated breeding values are dependent on the worm burden levels, and the $h^{2}$ estimates tend to increase at high levels of worm burden. Nevertheless, Marques et al. [38] estimated $h^{2}$ values for low- and high-burden worm environments that were not statistically different from each other (0.18 ± 0.02—low; 0.23 ± 0.02—high). In addition, the authors estimated a high genetic correlation of 0.87 (±0.03) between both the environments. According to the authors, the difference between both studies may be attributed to the animals’ age, in addition to the genotype by environment interaction.

In artificially infected animals, Aguerre et al. [28] estimated $h^{2}$ values for FEC of 0.14 (±0.04) and 0.35 (±0.08) for rams after the first and second artificial infections, respectively. Haehling et al. [22] estimated $h^{2}$ values for FEC of 0.25 (±0.18) and 0.46 (±0.19) after the first and second artificial challenges in Morada Nova lambs, respectively. For adult ewes, the same authors estimated $h^{2}$ values for FEC of 0.00 (±0.09) and 0.20 (±0.16) after the first and second challenges, respectively.

For the blood parameters, in a meta-analysis study performed by Medrado et al. [42], the authors estimated a global $h^{2}$ of 0.22 for HCT. After an artificial challenge among Morada Nova lambs, Haehling et al. [22] estimated $h^{2}$ values for PCV of 0.23 (±0.14) and 0.32 (±0.16) after the first and second artificial challenges, respectively. For adult ewes, the same authors estimated $h^{2}$ values for PCV of 0.13 (±0.11) and 0.37 (±0.18) after the first and second challenges, respectively. Aguerre et al. [28] estimated $h^{2}$ values for PCV of 0.24 (±0.05) and 0.18 (±0.06) for rams after the first and second artificial infections, respectively, and estimated an $h^{2}$ value for PCV of 0.12 (±0.08) for the naturally infected ewes. Marques et al. [38] estimated an $h^{2}$ for PCV of 0.25 (±0.06) using data from naturally infected Corriedale flocks. Oliveira et al. [43] estimated an $h^{2}$ of 0.30 (±0.06) for PVC in Santa Inês sheep breed naturally infected in Brazil. Bell et al. [32] estimated an $h^{2}$ for HCT of 0.27 (±0.12) for Merino sheep under a natural challenge with *H. contortus*. For the FAMACHA© score trait in a meta-analysis study performed by Medrado et al. [42], a global $h^{2}$ of 0.26 was estimated. Marques et al. [38] estimated an $h^{2}$ for FAMACHA© score of 0.10 (±0.02) in naturally exposed Corriedale sheep in Uruguay. Boareki et al. [27] estimated an $h^{2}$ of 0.07 (±0.05) for FAMACHA© in naturally infected Rideau Arcott sheep in Ontario, Canada. Oliveira et al. [43] estimated $h^{2}$ values of 0.21 (±0.04) for the FAMACHA© score in the Santa Inês sheep breed in Brazil.

Heritability estimates regarding the immune markers are not widely available in the literature. Bell et al. [32] estimated $h^{2}$ values of 0.37 (±0.16), 0.28 (±0.14), 0.33 (±0.16), and 0.10 (±0.09) for the lymphocyte, monocyte, eosinophil, and basophil levels in blood, respectively, in Merino sheep naturally challenged with *H. contortus*. Gutiérrez-Gil et al. [5] estimated an $h^{2}$ for the IgA levels of 0.19 (±0.05) at 60 days after deworming with natural exposure during grazing.

### 3.2. Genetic Correlations between Traits Measuring Resistance/Resilience and Production Traits

As indicated by Hayward [1], genetic correlations between resistance and production traits can vary from negative to positive, suggesting that increasing resistance may not change the production levels or may increase or decrease the production levels. Hayward [1] estimated a global genetic correlation of 0.10 between GIN resistance and performance traits in sheep. This estimate was not significantly influenced by the latitude, age, sex, statistical method, maternal effects, or parasite species. However, it was influenced by the type of infection and traits used to determine resistance and performance. According to the authors, when natural infection was used, the correlations between the resistance traits and performance traits were strongly positive, and under artificial infection conditions, the correlations were not significant. For the different resistance traits, including FEC, FAMACHA© scores, and immune markers, the global estimated genetic correlations with performance traits were ~0.1, ~0.3, and ~−0.15, respectively [1]. Finally, the genetic correlations between resistance and wool traits (~0.01) were smaller than those between resistance and daily weight gain (~0.10) and body condition score (~0.15) traits [1].

Genetic correlations between FEC and blood parameters have been estimated by relatively few authors. Medrado et al. [42] performed a meta-analysis, where the authors estimated genetic correlations of 0.70 between FEC and FAMACHA© scores and −0.48 between FEC and HCT. This indicates that a lower FEC is associated with lower FAMACHA© scores (red scores) and higher HCT levels (not anemic). Haehling et al. [22] estimated a significantly favourable genetic correlation for Morada Nova lambs between the FEC and PCV of −0.79 (±0.03) and −0.73 (±0.03) after the first and second artificial challenges, respectively. The authors also estimated phenotypic correlations under the same conditions of −0.64 (±0.05) and −0.67 (±0.04), indicating that lower FEC values are associated with greater PCV values. The genetic correlation between FEC and PCV for rams after the first artificial infection was 0.86 [22].

For the immune markers, Mucha et al. [39] pooled a genetic correlation of −0.40 (±0.05) for FEC and parasitism Ig, indicating lower Ig levels when higher egg counts were present. Gutiérrez-Gil et al. [5] estimated significant genetic correlations between FEC under natural exposure on the deworming day and IgA levels of 0.45 (±0.20) in adult ewes. According to the authors, young animals will normally present a negative genetic correlation between FEC and IgA levels because these animals control worm fecundity and grow through specific antibodies. However, the adult animals will have effective immune responses capable of controlling the number of parasites, which may justify the absence of a negative genetic correlation between FEC and IgA levels in adult animals.

Estimates of the genetic correlation between resistance and production traits are important and need to be estimated to optimize breeding programs to select for more resistant and productive animals. Based on the meta-analysis performed by Hayward [1], FEC showed a positive genetic correlation (about 0.10) with production traits. Medrado et al. [42] also estimated a favorable genetic correlation of −0.19 between FEC and BW in a meta-analysis.

In a meta-analysis study performed by Mucha et al. [39], the pooled estimate of the genetic correlation between milk yield and FEC was 0.17 (±0.35); however, this estimated value was not significant. Nevertheless, the range of genetic correlation identified by the authors between milk yield and FEC was from −0.21 (±0.26) to 0.63 (±0.01). Considering growth rates and FEC, Mucha et al. [39] pooled an estimated genetic correlation of −0.28 (±0.11), showing that small FECs are associated with a faster growth rate in animals. However, the individual estimates ranged from −0.68 to 0.57. In addition, the genetic correlation between FEC and body weight ranged from −0.90 to 0.34. According to the authors, these enormous variations may be explained by different factors, such as genotype–environment interactions, the fact that growth and immunity may split the available resources in the body, infection stage, nutritional factors, and linkage or pleiotropic effects between resistance and production traits. Safari et al. [40] published weighted mean estimates for genetic correlation obtained from the literature of 0.11, −0.03, −0.24, and −0.12 for FEC and birth weight, weaning weight, post-weaning weight, and adult weight, respectively, showing a favorable correlation between FEC and all the traits except birth weight. The estimated genetic correlations between growth rate and body weight are not similar across studies [12], showing a wide range that goes from negative to positive. The association between resistance/resilience and production traits is complex, and more understanding regarding the factors affecting this relationship is imperative to improve the genetic gains [39].

The trade-offs between resistance measured with FEC and production traits have been highlighted by a few studies [19,44]. However, according to Bisset et al. [44], when the resistant lines of sheep graze separately from the susceptible lines of sheep, the growth rates in the resistant lines were higher compared to when both the lines grazed together, which can be explained by the higher pasture burdens when both lines grazed together. Sallé et al. [19] stated that a few studies estimated favorable or non-significant genetic correlation between resistance and growth traits under natural grazing challenge, which was also identified in the other studies in this review [22,27,38]. Galyon et al. [11] observed that the Katahdin sheep that did not require deworming under a parasitic challenge were 5 kg heavier than the animals that needed treatment, indicating that selecting for a reduced FEC may not significantly affect the animals’ growth in a moderate challenge environment.

Estimates of the genetic correlations among production traits and blood parameters measuring GIN resistance are scarce in the literature. Oliveira et al. [43] estimated favourable genetic correlations of 0.59 (±0.15), −0.57(±0.12), and 0.73 (±0.11) between PCV and body weight, FEC, and BCS, respectively, for Santa Inês sheep under a natural exposure GIN in Brazil. These estimates indicate that higher PCV levels are associated with a greater BW, lower FEC, and improved BCS. The same authors also estimated favourable genetic correlations of −0.40 (±0.17), 0.77 (±0.09), and −0.59 (±0.11) between FAMACHA© score and BW, FEC, and BCS, respectively. According to Hayward [1], FAMACHA© scores showed a favourable genetic correlation (about 0.30) with production traits based on the meta-analysis results. Medrado et al. [42] estimated genetic correlations of 0.70 between FEC and FAMACHA© scores and −0.48 between FEC and HCT in a meta-analysis.

The meta-analysis studies performed by Hayward [1] and Mucha et al. [39] estimated genetic correlations between immune marker levels as traits measuring resistance to GINs and production traits. Hayward [1] estimated a negative genetic correlation (about 0.10) between immune markers measuring GIN resistance and production traits. Mucha et al. [39] pooled an estimated genetic correlation of −0.25 (±0.19) between BW and parasite-associated Ig; however, this estimate was not significant.

According to Hayward [1], the genetic correlations between resistance to GINs and production traits changed from strongly positive to around zero between 1980 and the present. According to the authors, this may have happened due to the publication of studies with larger datasets and more statistically significant results than studies that used smaller datasets and reported more non-significant results. The other possible explanations are the use of improved evaluation methods, and/or parasite adaptation to the climate or anthelmintics.

Regardless of whether breeding for resistance may reduce or maintain the same production levels, it is important to remember that diminishing the dependency on anthelmintic will reduce the costs associated with treatment, will decrease the antiparasitic residues in the environment and food, and can decelerate the development anthelmintic resistance [34]. In addition, the selection of genetically resistant animals can be considered a sustainable control strategy and will be more effective when combined with other control strategies [30,45].

### 3.3. Genetic Parameters for Anti-Carbohydrate Larval Antigen (CarLa)

An alternative novel phenotype that can be used for genetic selection is the level of anti-carbohydrate larval antigens (CarLa). This antigen is present on the surface of ensheathed L3 larvae of all the GIN species identified so far. Sheep infected with GIN will produce an IgA response against CarLa that can be measured and used to quantify the animal’s ability to build an immune response [18]. Shaw et al. [18] identified that the anti-CarLa IgA levels measured from the saliva collected with a buccal swab have an $h^{2}$ ranging between 0.19 (±0.11) to 0.39 (±0.18) and a favourable genetic correlation with FEC ranging from −0.72 (±0.22) to −0.34 (±0.13), where high anti-CarLa responders will have low fecal egg counts. According to the authors, anti-CarLa salivary IgA appears to be a candidate phenotype used to measure sheep’s resistance because it is heritable with a positive genetic correlation with FEC and associated with an increased live weight. Furthermore, when compared to the FEC, CarLa is easier and faster to measure, less invasive, and the animals will always have saliva in their body, which is not always the case with faeces [18]. This approach seems to be suited to places with different weather conditions [35]. Borkowski et al. [35] investigated the association between GIN infection and the salivary antibody response to CarLa in sheep raised in Ontario and concluded that the salivary CarLa level has the potential to be used in genetic selection to improve sheep’s resistance to GINs in the boreal climate of Ontario. However, further studies need to be performed to estimate the genetic parameters of CarLa level and their correlation with the production traits to better understand its viability in the future [1].

## 4. Selection for Resistance

### 4.1. Genetic Selection for Resistance

The strongest evidence that selection for resistance to GINs is possible and likely to remain in the population is the existence of naturally resistant breeds that emerged under natural selection [30]. Furthermore, other studies have shown that genetic improvement in sheep populations using only FEC as the selected trait is possible [30]. Eady et al. [34] rated the genetic and non-genetic methods, which alone had the greater impact on reducing the FEC. According to the authors, the most effective methods were genetic selection (69% reduction in the FEC), followed by protein supplementation (35% reduction in the FEC), and strategic drenching (28% reduction in the FEC). The gains obtained through genetic selection are cumulative and permanent. In addition, breeding for resistance is sustainable and can reduce the use of anthelmintics [30,34]. Burke et al. [37] identified that the need to deworm a flock was negatively correlated to the average daily gain, suggesting that more deworming is associated with a low average daily gain. However, it is important to consider genetic-by-environment interactions, when an animal selected under certain conditions is subject to another environment [30].

According to Burke et al. [37], the selection of rams with favourable estimated breeding values (EBVs) for a reduced FEC resulted in increased offspring resistance and resilience to GINs and fewer problems associated with parasitemia. Kemper et al. [45] used two lines (resistant and susceptible) of the Merino sheep breed to understand the host–parasite relationship. According to the authors, the resistant line has been selected for the past 15 years under natural grazing conditions and shows an annual genetic reduction of 2.7% for FEC. In this study, the resistant line had an average FEC of 134 eggs per gram (epg), which was just 18% of the average of the susceptible line (726 epg). According to Kemper et al. [45], the resistant line was able to reduce the adult worm numbers for both the studied worm species (*T. circumcincta* and *T. colubriformis*) and the fecundity of the *T. circumcincta* species, leading to a lower FEC.

Selecting rams under experimental conditions can also be effective in increasing the offspring’s resistance to GINs under natural grazing conditions. Aguerre et al. [28] showed that the offspring of resistant rams excreted, on average, half of the number of eggs when compared with the offspring of susceptible sires. This reduction was obtained by applying a 50% selection pressure. According to the authors, this reduction in the number of eggs in faeces is similar to the reduction observed 70 days after treatment with eprinomectin [28]. In addition, the ewes that shed more eggs into the environment were less common among the descendants of resistant rams, when compared to those of the susceptible rams, representing totals of 4% and 12% of the offspring, respectively. Fewer eggs shed in the environment reduces the pasture egg burden and increases the protective effect for the flock [28].

Genetic selection for resistance to GINs has been practiced by countries around the world for a long time. Some countries have made significant progress in developing genetic evaluations for resistance to GINs in sheep. Uruguay included resistance to GINs in their genetic evaluation system in 1994, using FEC records to select divergent lines for resistance in the Australian Merino and Corriedale animals [46]. This approach has been successful since the resistant lines have shown lower genetic trends for FEC when compared to the susceptible lines [46]. New Zealand has been estimating breeding values for resistance to GINs for more than two decades [44]. In the same way, Australia included resistance to GINs in their selection index a long time ago [47].

According to Ciappesoni et al. [46], selecting for resistance to GINs is a laborious job that requires the coordination, commitment, and contribution of several agencies, such as universities, farmers’ associations, and research institutes. Institutions must join forces in the measurement of traits, implementation of protocols, training, and knowledge transfer, especially in the initial stage of implementation [46], which can explain why some countries have not been able to include resistance to GINs in their genetic evaluation, even with the major economic impacts. Countries with systematic problems of GIN infection, such as Brazil, do not genetically evaluate the traits measuring GIN resistance in sheep, even for the main breeds such as for Santa Ines breed, which is a major Brazilian sheep breed [48]. Likewise, Canada does not include the resistance to GINs in its genetic evaluation, despite having records of anthelmintic resistance in the Ontario sheep population [49].

### 4.2. Genomic Selection for Resistance

Sallé et al. [19] used FEC phenotypic information and genomic information from 898 SNPs distributed along eight quantitative trait loci (QTL) significantly associated with GINs to perform a single-step genomic best linear unbiased prediction (ssGBLUP) of the genomic estimated breeding values (GEBV) and select the most resistant and most susceptible animals in a base population of Romane sheep. The authors identified phenotypic and genetic differences between the resistant and susceptible lines. After two generations, the resistant line differed from the base population, with a reduction of 0.62 in the phenotypic standard deviations for FEC in the first generation and 0.67 in the second generation of selection for the same trait. In addition, the GEBVs and EBVs for FEC showed a high correlation in the first (91%) and second (71%) infections. Sallé et al. [19] also stated that the resistant lines could maintain their response under chronic stress, but differences between families could be identified.

Some studies exploited the possibility of applying genomic information to improve the prediction accuracies in breeding programs [36,45,50]. The accuracies estimated by Pickering et al. [36] for FEC ranged from 0.18 to 0.71 for breeds of animals in the reference training set (set to train the marker effects). The prediction accuracies estimated across breeds may be less accurate if all the breeds are not well represented in both the training and validation population sets [36]. Similar results were discussed by Kemper et al. [45], showing that the improvement of genomic estimations requires all the breeds to be represented in the reference training population. In addition, Kemper et al. [45] stated that increasing the linkage disequilibrium (LD) between markers by increasing the SNP panel density may improve the genomic prediction accuracies, especially for the crossbreed populations. Despite the challenges, genomic selection can be beneficial in sheep breeding programs, as discussed by Salaris et al. [50] and Pickering et al. [36].

Uruguay implemented genomic information in its genetic evaluation program in 2021 [46]. Australia and New Zealand have also incorporated genomic information into their routine evaluations [47,51]. In Australia, the sheep industry has invested heavily in genomic selection and has fully incorporated genomics into their selection protocol [47,51]. As a result, Australian sheep breeders have seen significant genetic gains in both maternal and terminal sire production [51]. Additionally, Australian sheep breeders have seen genetic gains in hard-to-measure traits, including disease resistance [51]. Similar trends have been observed in New Zealand; in addition to growth and reproductive traits, they became to include health traits, such as worm resistance and facial eczema tolerance, into their evaluations [52].

## 5. Could Gastrointestinal Nematodes Overcome Sheep Resistance?

A question that arises when discussing improving sheep’s resistance to GINs is if the nematodes could overcome sheep resistance in the same way they overcame anthelmintic treatments [20]. There are several reasons why this may not be an issue. First, we need to consider that host resistance is not absolute and varies between animals [20]. Second, the resistance to GINs in sheep is a polygenic trait, and it is required that the parasites evade several mechanisms to break the host resistance, which is unlikely to happen [20,28]. Lastly, it is necessary to consider the parasite biology; the majority of the eggs are shed by susceptible hosts, which means that the genetic recombination of the future parasite population is determined by the susceptible hosts instead of the resistant ones. Additionally, only the worm population inside the host experiences the effects of selection; the parasites located in the pasture are not under selection to evade the biology of the host [20]. It is important to remember that parasites can evolve and overcome the used strategy. Thus, for a disease control plan to be effective and sustainable, it needs to combine different approaches [20,30].

## 6. Conclusions

Resistance to GINs is heritable, and sheep populations typically present sufficient genetic variability for it, making possible to genetically select the more resistant animals. Different countries have been successfully selecting for resistance to GINs for decades. Recently, these countries have incorporated genomic information into their genetic evaluation systems, which led to higher breeding value prediction accuracies and accelerate genetic gains. Improving the flocks to become more resistant to GINs is one way to decrease the reliance on anthelmintic drugs, cut the treatment expenses, decrease the environmental and food residues, and possibly slow the development of anthelmintic resistance. Raising more resistant animals is more sustainable and will be more effective when combined with the other control strategies.

## Figures and Tables

**Table 1:** Heritability estimates for traits measuring resistance to gastrointestinal nematodes from different studies.

Trait	Heritability Estimates	Reference
Fecal egg counts	0.17 *	[42]
	0.14 * (±0.04)	[39]
	0.29 * (±0.03)	[39]
	0.27 * (±0.02)	[40]
	0.19 (±0.03)	[43]
	0.10 (±0.04)	[27]
	0.07 (±0.05)	[27]
	0.12 (±0.04)	[27]
	0.12 (±0.04)	[5]
	0.09 (±0.03)	[5]
	0.21 (±0.02)	[16]
	0.38 (±0.03)	[16]
	0.20 (±0.03)	[12]
	0.19 (±0.03)	[12]
	0.37 (±0.03)	[12]
	0.25	[12]
	0.36	[12]
	0.14 (±0.04)	[28]
	0.35 (±0.08)	[28]
	0.25 (±0.18)	[22]
	0.46 (±0.19)	[22]
	0.00 (±0.09)	[22]
	0.20 (±0.16)	[22]
Hematocrit	0.22 *	[42]
	0.27 (±0.12)	[22]
Packed cell volume	0.23 (±0.14)	[22]
	0.32 (±0.16)	[22]
	0.13 (±0.11)	[22]
	0.37 (±0.18)	[22]
	0.24 (±0.05)	[28]
	0.18 (±0.06)	[28]
	0.12 (±0.08)	[28]
	0.25 (±0.06)	[38]
	0.30 (±0.06)	[43]
FAMACHA© score	0.26 *	[42]
	0.10 (±0.02)	[38]
	0.07 (±0.05)	[27]
	0.21 (±0.04)	[43]
Lymphocyte blood levels	0.37 (±0.16)	[22]
Monocyte blood levels	0.28 (±0.14)	[22]
Eosinophil blood levels	0.33 (±0.16)	[22]
Basophil blood levels	0.10 (±0.09)	[22]
Immunoglobulin A levels	0.19 (±0.05)	[5]

* Heritability estimated in meta-analysis and/or literature review. FAMACHA©: FAffa MAlan CHArt.

## Data Availability

The data used in this manuscript may be made available by a reasonable request sent to the corresponding author.
